# The first appearance deceives many: Isolated RV infarct masquerading as an anterior STEMI

**DOI:** 10.1002/ccr3.3061

**Published:** 2020-06-23

**Authors:** Umar Ismail, Olga Toleva, Davinder S. Jassal

**Affiliations:** ^1^ Section of Cardiology Department of Internal Medicine Max Rady College of Medicine Rady Faculty of Health Sciences University of Manitoba Winnipeg MB Canada; ^2^ Department of Radiology Max Rady College of Medicine Rady Faculty of Health Sciences University of Manitoba Winnipeg MB Canada

**Keywords:** EKG, myocardial infarction, right ventricle

## Abstract

Although ST‐segment elevation in the precordial leads on an EKG is highly suggestive of occlusion of the left anterior descending artery, the pattern can also result from isolated right ventricular (RV) infarction.

## Graphical abstract

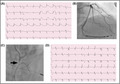

## 1 CLINICAL QUESTION

Is precordial ST elevation on an EKG always due to occlusive disease of the left anterior descending artery?

**FIGURE 1:**
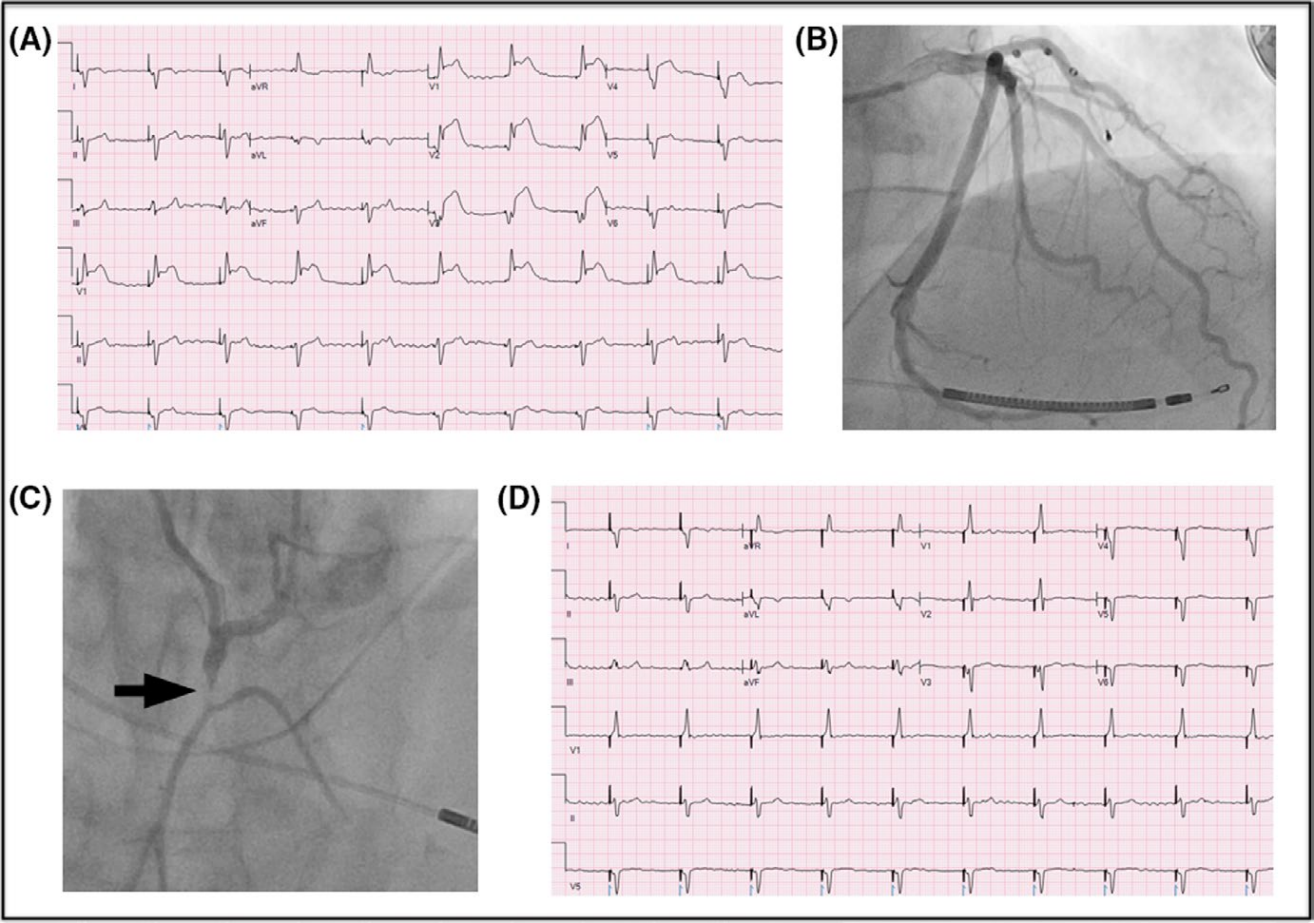
A, 12‐lead EKG on presentation demonstrating ST‐segment elevation in the precordial leads in the setting of biventricular pacing. B, Cardiac catheterization in the right anterior oblique view demonstrating no evidence of significant obstructive coronary artery disease of the left dominant system (Video S1). C, Cardiac catheterization of the culprit RCA in the left anterior oblique view demonstrating acute occlusion of its proximal segment (Video S2). D, 12‐lead EKG immediately following PCI of the RCA

A 55‐year‐old man with a history of resolved tachycardia‐induced cardiomyopathy after AV node ablation and cardiac resynchronization therapy defibrillator (CRT‐D) implantation presented with chest pain. The initial EKG demonstrated biventricular pacing with ST elevation in leads V1 to V4 (Figure 1A). Coronary angiography revealed a left dominant system without occlusive disease (Figure 1B). The nondominant RCA had a 90% stenosis in its proximal segment with thrombolysis in myocardial infarction (TIMI) I flow (Figure 1C). The RCA was revascularized with a 2.5 × 16 mm Promus Premier drug‐eluting stent. Following restoration of TIMI III flow, the ST elevation in the precordial leads resolved (Figure 1D).

Accounting for 3% of all infarctions, RV myocardial infarction (MI) results from occlusion of the RCA proximal to the RV marginal branches.^1^ Two previous case reports have reported isolated precordial ST elevation secondary to nondominant RCA occlusion.^1^, ^2^ Although the previous case reports had no CRT device in situ, the presence of biventricular pacing could have affected the mean vector of electrical forces in our case. Although precordial ST elevation is highly indicative of left anterior descending artery occlusion, occlusion of the RV marginal branch of a dominant or nondominant RCA should be entertained in the differential diagnosis.

## CONFLICT OF INTEREST

UI, OT, and DJ declare no conflict of interest to declare.

## AUTHOR CONTRIBUTION

UI, OT, and DJ: Contributed to the writing and approval of the final manuscript.

## Supporting information

Video S1

Video S2
